# Estimates of linkage disequilibrium and effective population size in rainbow trout

**DOI:** 10.1186/1471-2156-10-83

**Published:** 2009-12-14

**Authors:** Caird E Rexroad, Roger L Vallejo

**Affiliations:** 1 USDA/ARS National Center for Cool and Cold Water Aquaculture, Leetown, West Virginia, 25430, USA

## Abstract

**Background:**

The use of molecular genetic technologies for broodstock management and selective breeding of aquaculture species is becoming increasingly more common with the continued development of genome tools and reagents. Several laboratories have produced genetic maps for rainbow trout to aid in the identification of loci affecting phenotypes of interest. These maps have resulted in the identification of many quantitative/qualitative trait loci affecting phenotypic variation in traits associated with albinism, disease resistance, temperature tolerance, sex determination, embryonic development rate, spawning date, condition factor and growth. Unfortunately, the elucidation of the precise allelic variation and/or genes underlying phenotypic diversity has yet to be achieved in this species having low marker densities and lacking a whole genome reference sequence. Experimental designs which integrate segregation analyses with linkage disequilibrium (LD) approaches facilitate the discovery of genes affecting important traits. To date the extent of LD has been characterized for humans and several agriculturally important livestock species but not for rainbow trout.

**Results:**

We observed that the level of LD between syntenic loci decayed rapidly at distances greater than 2 cM which is similar to observations of LD in other agriculturally important species including cattle, sheep, pigs and chickens. However, in some cases significant LD was also observed up to 50 cM. Our estimate of effective population size based on genome wide estimates of LD for the NCCCWA broodstock population was 145, indicating that this population will respond well to high selection intensity. However, the range of effective population size based on individual chromosomes was 75.51 - 203.35, possibly indicating that suites of genes on each chromosome are disproportionately under selection pressures.

**Conclusions:**

Our results indicate that large numbers of markers, more than are currently available for this species, will be required to enable the use of genome-wide integrated mapping approaches aimed at identifying genes of interest in rainbow trout.

## Background

The use of molecular genetic technologies for broodstock management and selective breeding of aquaculture species is becoming increasingly more common with the continued development of genome tools and reagents for species of interest [1]. Rainbow trout are the most widely produced salmonid in the US, attracting significant interest due to their economic impacts as an aquaculture species and on sport fisheries, and as a model research organism for studies related to carcinogenesis, toxicology, comparative immunology, disease ecology, physiology and nutrition [2]. To this end several international laboratories have produced genetic maps for this species to aid in the identification of loci affecting phenotypes of interest. These maps primarily include amplified fragment length polymorphisms (AFLPs) and microsatellites [3-9] and have resulted in the identification of many quantitative/qualitative trait loci (QTL) affecting phenotypic variation in traits associated with albinism, disease resistance, temperature tolerance, sex determination, embryonic development rate, spawning date, condition factor and growth [10-19]. In spite of these efforts, the elucidation of the precise allelic variations and/or genes underlying phenotypic diversity has yet to be achieved in this species having low marker densities and lacking a whole genome reference sequence.

Experimental designs which integrate complex segregation analyses with linkage disequilibrium (LD) approaches facilitate the discovery of genes affecting important traits [20-22]. To this end, the extent of LD has been characterized for humans and several agriculturally important livestock species including cattle, sheep, chickens, and pigs. Farnir *et al*. [20] genotyped 284 autosomal microsatellite markers on 581 dutch black-and-white dairy cattle to construct a whole genome LD map (excluding the sex chromosomes) spanning 2702 cM. Estimations of LD between syntenic loci using Lewontin's normalized *D*' [23] revealed large blocks of LD spanning tens of centiMorgans including values of 50% for markers <5 cM and decaying to 16% for distances of 50 cM. The value of *D*' between non-syntenic loci was estimated to be 12%. Vallejo et al. [24] selected distantly related animals to quantify the level of genetic diversity in United States Holstein cattle. While only 23 Holstein bulls were genotyped with 54 microsatellite loci that spanned most of the autosomal genome, extensive LD was detected in the United States Holstein population in agreement with the findings of Farnir et al. [20]. In 2002, McRae *et al*. [25] observed similar results by genotyping 90 microsatellites from 10 chromosomes on 276 progeny from Coopworth sheep, estimating *D*' values of 34.3% for marker distances <60 cM, 12.4% for syntenic markers >60 cM, and 12.4% for non-sytenic loci. In 2005 Heifetz *et al*. [26] reported that significant LD was only observed for loci < 5 cM apart in a commercial layer chicken population. In 2006 Harmegnies *et al*. [27] characterized LD in two commercial pig populations of 33 and 44 unrelated individuals by genotyping 29 and 5 microsatellite markers on two chromosomes, respectively. Estimates of *r*^2 ^(squared correlation coefficient) revealed significant LD only for loci < 1 cM apart. More recently, Du *et al*. [28] and McKay *et al*. [29] evaluated LD in pigs and cattle, respectively, by genotyping syntenic single nucleotide polymorphisms (SNPs) and observing significant LD for marker pairs < 3 cM apart for pigs and 0.5 Mb for cattle. All of these results indicate that large numbers of markers from high-density maps are required to identify genes of interest using whole genome association studies in these species.

The USDA/ARS National Center for Cool and Cold Water Aquaculture (NCCCWA) has established a breeding program for rainbow trout, the use of molecular genetic technologies in this program is expected to enhance capabilities for selective breeding of important aquaculture production traits. To this end we have worked within international collaborations to develop genomic tools and technologies for rainbow trout [5,8,9,30-46] while initiating and characterizing our broodstock population with respect to genetic and phenotypic variation relevant to aquaculture production [47-57]. Our approaches include genetic linkage and LD mapping for the identification of QTL affecting traits of economic importance which will permit the development of marker/gene assisted selection strategies [58,59] and eventually genomic selection [60]. Characterizing the extent of LD in the NCCCWA rainbow trout broodstock population will support the use of integrated mapping approaches and facilitate the identification of genes affecting traits of interest by determining the marker densities required to conduct genome association scans. The NCCCWA broodstock population is closely related to commercial germplasm [49], therefore our findings also have the potential to impact industry selective breeding programs. In addition to LD we have evaluated effective population size (*Ne*) in an effort to characterize the true breeding size of our population. This estimate of *Ne *should be considered when making decisions concerning selection pressure. To this end, we characterized extent of LD by genotyping 96 unrelated individuals with 49 markers spanning four chromosomes.

## Results

Genotypes were obtained for a total of 49 microsatellites on chromosomes OMY13 (n = 6), OMY14 (n = 20), OMY17 (n = 8), and OMYSex (n = 15). Genotyping success rate was 95.5% with 82 to 96 animals scored for each marker. The number of alleles per marker, marker heterozygosity, PIC, Allelic Diversity, and exact tests for departure from HWE proportions are reported in Table 1. The number of alleles per marker averaged 10.6 and ranged from 3 to 24. Marker heterozygosities ranged from 28.1% to 94.8% with an average of 69.8%. Polymorphism Information Content and Allelic Diversity ranged from 0.385 to 0.936 and 0.425 to 0.94, with averages of 0.745 and 0.773, respectively. A total of 19 loci had significant departure from *HWE *at *P *< 0.05, consisting of 3 markers from OMY13, 9 from OMY14, 2 from OMY17, and 5 from OMYSex.

**Table 1:** Exact test for Hardy-Weinberg equilibrium for microsatellite loci typed in 96 unrelated fish from the NCCCWA rainbow trout selective breeding program.

Locus	GenBank Acc. or reference	OMY	Posit. Kos cM^1^	Number Alleles	PIC^2^	Hetero- zygosity	Allelic Diversity	*χ*^2^	DF	Pr > *χ*^2^	Exact *P*^3^
OMM3006	G73806	13	39.9	13	0.844	0.802	0.858	127.6	78	0.0003	0.0615
OMM5092	CA348764	13	39.9	5	0.705	0.708	0.746	20.7	10	0.0231	0.0152
OMY27DU	[4]	13	44.6	5	0.575	0.594	0.618	5.7	10	0.8402	0.7166
OMM1670	BV212162	13	44.6	16	0.889	0.844	0.898	242.4	120	<.0001	0.0072
CA341677	CA341677	13	44.6	6	0.544	0.604	0.590	99.5	15	<.0001	0.2147
OMM1687	BV212173	13	45.7	3	0.390	0.417	0.501	7.2	3	0.0659	0.0325
Oneu8	U56708	14	22.2	6	0.596	0.583	0.654	5.8	15	0.9824	0.8181
OMM1312	G73552	14	49.2	16	0.882	0.885	0.891	125.5	120	0.3477	0.1418
OMYRGT2TUF	AB087587	14	50.7	9	0.724	0.750	0.748	34.9	36	0.5218	0.6786
OMM1596	BV212112	14	53.9	13	0.857	0.844	0.871	98.9	78	0.0552	0.0068
OMM5271	BV211986	14	58.3	11	0.806	0.802	0.827	53.6	55	0.5286	0.4101
OmyRGT41TUF	[4]	14	58.3	16	0.876	0.854	0.885	114.9	120	0.6136	0.3824
Ogo1UW	AF007827	14	58.3	5	0.620	0.635	0.679	98.9	10	<.0001	0.1218
OMM3089	BV718454	14	62.5	4	0.612	0.760	0.679	6.1	6	0.4098	0.3408
OMM1447	BV079591	14	63.4	22	0.936	0.823	0.940	237.6	231	0.3696	0.0017
OMM3115	AB162343	14	63.4	12	0.843	0.490	0.859	219.2	66	<.0001	<.0001
BHMS429	AF256719	14	64.3	12	0.890	0.885	0.898	50.5	66	0.9214	0.9010
OMYFGT5TUF	[4]	14	64.3	10	0.569	0.604	0.620	40.8	45	0.6515	0.3260
BHMS185	AF256675	14	72.9	3	0.454	0.583	0.546	2.8	3	0.4239	0.2933
OMM1415	BV722101	14	75.0	11	0.793	0.719	0.811	76.0	55	0.0317	0.0233
OMM1356	BV005148	14	79.1	4	0.385	0.281	0.432	138.6	6	<.0001	<.0001
CR374305	CR374305	14	81.2	13	0.790	0.646	0.809	439.9	78	<.0001	<.0001
OMM1467	BV079662	14	82.7	12	0.866	0.906	0.878	79.2	66	0.1271	0.1546
OMM1643	BV212150	14	86.5	11	0.764	0.750	0.785	89.7	55	0.0022	0.0354
OMM5143	BV211872	14	106.2	11	0.828	0.427	0.846	271.4	55	<.0001	<.0001
OMM3044	BV722031	14	130.0	8	0.739	0.396	0.775	242.4	28	<.0001	<.0001
OMM1712	BV212193	17	81.7	13	0.855	0.823	0.868	67.9	78	0.7859	0.5445
CA367675	CA367675	17	84.8	12	0.851	0.917	0.864	51.7	66	0.9017	0.8540
OMM5043	CA349167	17	89.5	8	0.795	0.750	0.820	34.5	28	0.1836	0.1205
OMM3126	BV683039	17	90.3	18	0.904	0.833	0.911	144.1	153	0.6841	0.1027
OMM1437	BV722116	17	91.3	15	0.897	0.865	0.904	105.6	105	0.4648	0.1512
OMM1357	BV005149	17	105.4	17	0.867	0.833	0.877	207.1	136	<.0001	0.0305
OMM5227	BX301679	17	107.8	7	0.697	0.510	0.738	91.2	21	<.0001	<.0001
CA041953	CA041953	17	136.3	3	0.420	0.458	0.476	2.0	3	0.5711	0.4895
OMM1026	AF346683	sex	0.0	15	0.880	0.896	0.890	123.9	105	0.1007	0.5136
OMM1461	BV079604	sex	17.7	5	0.637	0.625	0.683	5.1	10	0.8870	0.7533
OMYRGT28TUF	AB087599	sex	21.1	15	0.830	0.760	0.846	172.6	105	<.0001	0.1674
OMM1000	AF346664	sex	21.1	3	0.385	0.417	0.425	0.5	3	0.9170	0.9396
OMM5031	CA349143	sex	29.7	9	0.722	0.865	0.753	59.8	36	0.0076	0.0005
OMM5032	CA349143	sex	29.7	11	0.842	0.844	0.857	94.7	55	0.0007	0.0895
BX076085	BX076085	sex	35.6	12	0.868	0.646	0.879	156.8	66	<.0001	<.0001
OMM1372	BV005159	sex	36.0	6	0.560	0.323	0.604	98.1	15	<.0001	<.0001
OMM1212	BV722077	sex	44.3	11	0.794	0.781	0.813	61.9	55	0.2421	0.2160
OMM1443	BV079588	sex	44.3	13	0.873	0.896	0.884	94.5	78	0.0984	0.0511
OMM3109	BV718471	sex	46.2	13	0.754	0.677	0.771	101.8	78	0.0363	0.0754
OMM1456	BV079600	sex	46.9	7	0.691	0.729	0.736	14.6	21	0.8447	0.7070
OMM1118	AF375026	sex	47.7	24	0.921	0.948	0.925	314.2	276	0.0564	0.1749
OMM1405	BV722096	sex	51.3	11	0.817	0.594	0.835	141.1	55	<.0001	<.0001
OMM1665	BV212292	sex	58.5	17	0.881	0.656	0.890	325.0	136	<.0001	<.0001

^1^Microsatellite loci position in the NCCCWA rainbow trout genetic map [9].

^2^Polymorphic information content (PIC).

^3^Exact *P*-value estimated using 10,000 permutations with SAS Proc Allele [70].

**Figure 1:**
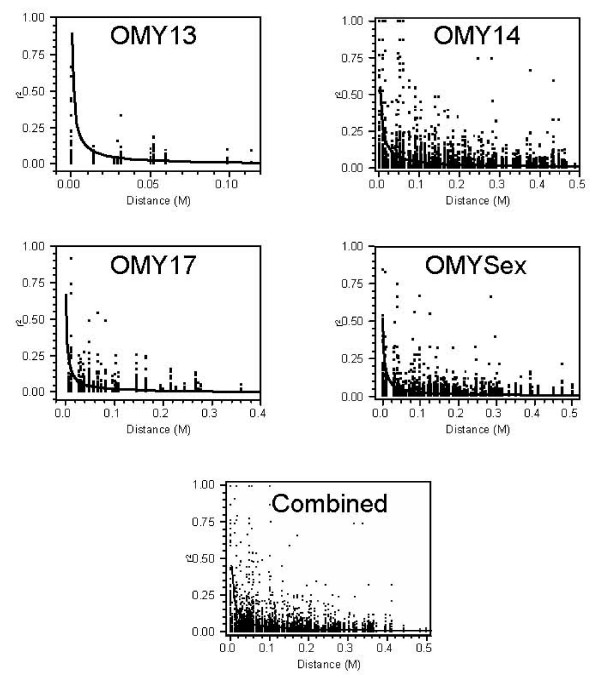
**Decline of linkage disequilibrium (*r*^2^) with distance (recombination rate in Morgans) for chromosomes 13, 14, 17 and Sex**. The estimates of *r*^2 ^for pairs of markers were adjusted for experimental sample size *1/n*, were *n *is the chromosome sample size (*n *= 192). The predicted *LD *value plot (filled non-linear curve) was estimated fitting the equation *LD*_*ij *_= 1/(1+*kb*_*j*_*d*_*ij*_)+*e*_*ij *_performing non-linear modeling with JMP^® ^Genomics 3.1 (SAS Institute Inc., Carey, NC, 2007). Here, *LD*_*ij *_is the observed *LD *for marker pair *i *in chromosome *j*, *d*_*ij *_is the distance in Morgans for marker pair *i *in chromosome *j, b*_*j *_is the estimate of effective population size for chromosome *j*, and the constant *k *= 2 for sex chromosome and *k *= 4 for autosomes.

LD for syntenic loci was estimated for each individual chromosome and the four chromosomes combined (Figure 1), the genome-wide estimate for extent of significant LD (*r*^2 ^> 0.25) was about 2 cM. The non-linear modeling of LD decline using the Sved (1971) equation indicates that the extent of significant LD decays sharply with physical distance. It also indicates that significant LD effects (i.e., *r*^2 ^≥ 0.25) are not expected beyond 5 cM.

**Figure 2:**
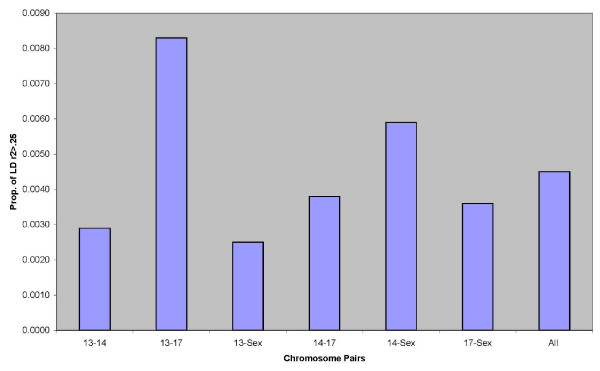
**Proportion of markers pairs with significant extent of LD (*r*^2 ^≥ 0.25)**. All marker pairs were evaluated in addition to pairwise combinations of all non-syntenic loci.

The LD for non-syntenic loci was estimated for each chromosome pairing and all pairings of non-syntenic loci, the proportion of pairings having (*r2 *> 0.25) is shown in Figure 2. Clearly, the proportion of significant LD (*r*^2 ^≥ 0.25) among non-syntenic loci was quite low

Effective population sizes were estimated for each chromosome and the four chromosomes as shown in Table 2. The estimated average effective population size was *N*_*e *_= 145 with an average *N*_*e*_/*N *ratio of 0.45.

**Table 2:** Effective population size (*N*_*e*_) estimated from linkage disequilibrium^1 ^by fitting nonlinear regression model^2 ^in a rainbow trout broodstock population^3^.

						Confidence interval		
							
Chromosome	Number of marker pairs	Average intermarker distance (cM)	Genome coverage (cM)		Standard error	-95%	+95%	
13	421	1.16	5.80	178.56	75.38	65.77	448.56	0.56
14	8817	5.67	107.80	75.51	5.62	65.24	87.14	0.24
17	1499	7.80	54.60	122.08	19.16	89.45	162.23	0.38
sex	5813	4.18	58.50	203.35	16.94	170.92	240.54	0.64
**Total**	16550.00	18.81	226.70	579.50	117.10	391.38	938.47	1.82

**Mean**	4137.50	4.70	56.68	144.88	29.27	97.85	234.62	0.45

**SD**				57.40	31.30	50.01	155.77	0.18

^1^Pairwise Linkage disequilibrium (LD) was estimated using the ALLELE procedure of the software package SAS^®^, version 9.3.1 (SAS Institute 2007).

^2^The nonlinear regression model was fitted using JMP^® ^Genomics 3.1 (SAS Institute Inc., Carey, NC, 2007),

Where  is LD measure adjusted for chromosome sample size *n*, for marker pair *i *at recombination rate *c*_*i *_(in Morgans). The constant *k *had values of *k *= 2 for sex chromosome and *k *= 4 for autosomes. The *c*_*i*_'s were estimates of recombination rate from two-point linkage analysis [9]. First, the *e*_*i *_residuals were estimated by non-linear fitting of the above model with JMP^® ^Genomics 3.1 (SAS Institute Inc., Carey, NC, 2007). Then, the parameters *α *and *β *were estimated iteratively by least squares; in this model .

^3^Unrelated individuals (*n *= 96) representing the 2005/2006 brood classes were genotyped with 49 microsatellite markers.

^4^Number of potential breeders (*N *= 320).

## Discussion

The extent of LD in the NCCCWA selective breeding program was evaluated by genotyping 49 microsatellites from four chromosomes on a total of 96 unrelated individuals from the 2005 (n = 43) and 2006 (n = 53) brood classes. As a result of an evolutionarily recent autopolyploid genome duplication event [61], many microsatellite markers for rainbow trout amplify two loci which can be scored independently and placed on genetic maps [4]. Although medium density genetic maps based on microsatellites including duplicated markers have been constructed [6,9], they are problematic for use in population genetic analysis such as whole genome association studies as alleles from the two loci often have overlapping and/or identical allele sizes. Thus the number of loci available for use in whole genome association studies is much less than what is available for segregation analyses. Only evaluating single locus markers resulted in observation of a 6 cM region of OMY13 with 6 loci, a 108 cM region of OMY14 with 20 loci, a 55 cM region of OMY17 with 8 loci, and a 58 cM region of OMYSex with 15 loci. Overall, 227 cM of the 2927 cM genome was represented in this study. Of interest is the 55 cM region of OMY14 spanning the region from 75-130 cM where 6/7 loci depart from *HWE*. It is possible that these loci are under selection in the NCCCWA broodstock population which includes selective pressure for disease resistance and growth.

The level of LD between syntenic loci decayed rapidly at distances greater than 2 cM which is similar to observations of LD in other agriculturally important species including cattle [20], sheep [25], pigs [27,28] and chickens [26]. However, significant LD was also observed up to 40 cM as reported by Farnir *et al*. [20] and McRae *et al*. [25] (Figure 1). The average proportion of significant LD (*r*^2 ^> 0.25) between non-syntenic loci was under .005, however OMY13 and OMY17, which have a common ancestor chromosome resulting from the salmonid whole genome duplication event, showed a significantly higher proportion of significant LD.

We acknowledge that the LD range reported here may be up-biased because evidences for structure and admixture have been shown in the NCCCWA Broodstock population [62] and we used unrelated individuals from 2005 and 2006 brood years that are being improved for disease resistance and production traits, respectively, in the NCCCWA selective breeding program.

As the NCCCWA selective breeding program continues throughout successive generations, we expect that the effective population size will continue to decrease. Our estimate of N_e _= 145 (Table 2) based on genome wide LD indicates that this population will respond well to high selection intensity as it has for a single generation of breeding for resistance to the causative agent of bacterial cold-water disease [50]. Using this estimate we can observe the effects of selection and identify correlations with the effects of inbreeding, usually first observed in reproductive success. However, the range of effective population size based on individual chromosomes was 75.51 - 203.35, possibly indicating that suites of genes on each chromosome are disproportionately under selection pressure. This also establishes the need to develop whole genome applications for these species for studies of LD. Although *F*-statistics have been used to estimate population genetic parameters [49], characterization of LD will also provide valuable information about population substructure and should be a factor in developing strategies aimed at the identification of associations between markers and QTL.

We chose to evaluate the extent of LD by genotyping representatives of each year class a single generation after the population was founded by mixing germplasm from multiple sources. Our resulting characterization of LD not only provides information about population structure, but serves to document the degree of genetic diversity used to initiate the broodstock program. We expect our estimates of Ne to be higher than populations with limited genetic diversity and no introduction of new germplasm in recent past generations.

The estimated *N*_*e*_/*N *ratio of 0.45 in this study is between the ranges of estimates reported in this species in previous studies [63-65]. Other studies in salmonids consistently reported that the variance in reproductive success is the key factor to reduce *N*_*e*_/*N *in salmon populations [64,66].

Characterizing the extent and distribution of LD helps to determine the required marker density for LD mapping and genomic selection as they both require markers to be in LD with QTL. Our observation of significant syntenic LD at distances over 2 cM has implications for designing genome wide association studies in this population and on this species. The sex averaged map is roughly 3000 cM long; therefore 1500 markers are required to identify loci of interest. The male map having 2500 cM would require 1125 markers; the female map having 4300 cM would require 2150 markers. Currently about 1800 microsatellite markers are available for genome analyses in trout. However, we must take into consideration that: 1) roughly 33% are duplicated; 2) on average 70% are informative based on estimates of heterozygosity; and 3) these markers are not necessarily spaced at regular intervals throughout the genome.

## Conclusions

To effectively conduct whole genome association studies the number of available markers for rainbow trout must be increased. Whereas genotyping microsatellites can be very expensive and time consuming process, it is likely that a marker system that enables high-throughput genotyping protocols such as single nucleotide polymorphisms will be the basis for LD mapping and genomic selection studies in the near future.

## Methods

### Germplasm

The NCCCWA rainbow trout selective breeding program was initiated in 2002 through 2004 with the acquisition of fish from Troutlodge, Inc. (Sumner, WA), the Donaldson strain from the University of Washington (Seattle, WA), the House Creek strain from the College of Southern Idaho (Twin Falls, ID), and the Shasta strain from the Ennis National Fish Hatchery, (Ennis, MT) [49]. To date, selective breeding for increased aquaculture production efficiency has been conducted by evaluating and selecting for growth traits on even years and resistance to challenge by the bacterial pathogen *Flavobacterium psychrophilum *on odd years [47,50]. To identify individuals that could best represent the genetic variation contained within this broodstock population, we identified 96 unrelated individuals (no siblings or half-siblings) from the 2005 and 2006 brood year classes (n = 43 and 53, respectively) to represent the 144 and 177 fish that were actually used to produce select matings. The 2005 year class, part of our odd year selection for disease resistance, is the first generation after the 2003 founder population. The 2006 year class, part of our even year selection for growth, is the second generation after the founder population in 2002. However, a significant contribution of new germplasm was also introduced in 2004. Fin clips from anesthetized fish were collected for DNA extraction as outlined in a protocol approved by the NCCCWA IACUC, number 025-1-26-05. The extraction protocols followed the phenol-chloroform method described in Sambrook and Russell [67]. DNA samples were quantified by spectrophotometer (Beckman DU 640, Beckman Instruments, St. Louis, MO, USA) and diluted to a concentration of 12.5 ng/ul for PCR.

### Genotyping

Microsatellite markers from four chromosomes (n = 49) were selected from the NCCCWA genetic map [9] for genotyping the DNA panel of 96 fish representing NCCCWA broodstock (Table 1). This included markers mapped to the sex chromosome (OMYSex), chromosome 14 having the largest linkage group in cM (OMY14), and paralogous regions of chromosomes 13 and 17 (OMY13, OMY17) as defined by duplicated loci resulting from an evolutionarily recent genome duplication event [4,9,61]. Only single locus markers were utilized. Markers were either genotyped using the tailed protocol [68] or by direct fluorescent labeling (with FAM, HEX, or NED) of the forward primer according to manufacturer protocols [[69] USA]. Primer pairs were obtained from commercial sources (forward primers labeled with FAM or HEX from Alpha DNA, Montreal, Quebec, Canada, or NED from ABI, Foster City, CA, USA). PCR reactions consisted of 12 *μ*l reaction volumes containing 12.5 ng DNA, 1.5-2.5 mM MgCl_2_, 1.0 *μ*M of each primer, 200 *μ*M of dNTPs, 1× manufacturer's reaction buffer and 0.5 units Taq DNA polymerase. Thermal cycling consisted of an initial denaturation at 95°C for 15 min followed by 30 cycles of 95°C for 1 min, annealing temperature for 45 s, 72°C extension for 45 s and a final extension at 72°C for 10 min. PCR products were visualized on agarose gels after staining with ethidium bromide. Markers were grouped in combinations of two or three markers based on differences in fluorescent dye color and amplicon size. Three *μ*l of each PCR product was added to 20 *μ*l of water, 1 *μ*l of the diluted sample was added to 12.5 *μ*l of loading mixture made up with 12 *μ*l of HiDi formamide and 0.5 of Genscan 400 ROX internal size standard. Samples were denatured at 95°C for 5 min and kept on ice until loading on an automated DNA sequencer ABI 3730 DNA Analyzer (ABI, Foster City, CA, USA). Output files were analyzed using GeneMapper version 3.7 (ABI, Foster City, CA, USA), formatted using Microsoft Excel and stored in Microsoft Access database.

### Analysis

The initial dataset for analysis included marker genotype data for 49 microsatellite loci typed on 96 unrelated individuals. These marker genotype data were analyzed to estimate frequency of alleles per marker using the ALLELE procedure of software package SAS^®^, version 9.3.1 [70]. Within each marker, alleles with frequency ≤ 0.01 were merged to minimize the up biasing effect of rare alleles on LD estimates. Then, the marker alleles initially recorded in size of fragments were recoded into a consecutive-numbered allele system using the computer program RECODE [71]. This dataset with recoded marker genotypes were divided into files including marker loci corresponding to each of the four linkage groups analyzed in this study. Subsequently, these recoded marker genotype data were used in haplotype reconstruction and linkage disequilibrium analysis.

### Haplotype reconstruction

For each linkage group, the most likely haplotype configuration for each individual was estimated using the software package PHASE, version 2.1, following procedures described by [72] and [73]. Briefly, this is a statistical method for inferring haplotypes from unphased genotype data for a sample of "unrelated" individuals from a population; population haplotype frequencies are assumed under Hardy-Weinberg equilibrium (*HWE*) but PHASE has proven robust to deviations from HWE, the effect of population structure and moderate amounts of recombination. The estimated haplotype data was formatted for subsequent analysis with the ALLELE procedure of the software package SAS^®^, version 9.3.1 [70]. The haplotype data was formatted into SAS ALLELE procedure format (option TALL for data input format) using a Java script (Haplo_2SAS.jar) written by Christopher Schmitt. This script is available for academic use upon request to RLV. This formatted dataset was used in linkage disequilibrium analysis.

### Hardy-Weinberg equilibrium

The *HWE *analysis for each of the 49 loci typed in 96 unrelated individuals was performed using the ALLELE procedure of the software package SAS^®^, version 9.3.1 [70] using the option TALL for data input format. The input data were the most likely haplotypes reconstructed for each individual using the computer program PHASE and reformatted into SAS format as outlined above. The exact test for *HWE *for each microsatellite loci (exact *P-*value) was estimated using 10,000 permutations.

### Linkage disequilibrium between syntenic loci

The LD analysis was performed between marker pairs within each linkage group using the ALLELE procedure of software package SAS^®^, version 9.3.1 [70]. We used the option TALL for data input format, and the HAPLO = GIVEN option which indicates that the haplotypes have been observed, and thus the observed haplotype frequencies were used in the LD test statistic and measures.

The linkage (or gametic) disequilibrium coefficient *D*_*uv *_between two alleles from marker loci *M *and *N*, respectively, was estimated using this expression [74],

Where *p*_*uv *_is probability that an individual receives the haplotype *M*_*u*_*N*_*v *_for marker loci *M *and *N*, *p*_*u *_is the probability of the *u *allele, and *p*_*v *_is the probability of the *v *allele.

The ALLELE procedure calculates the maximum likelihood estimates, , of the LD coefficient between a pair of alleles at different markers. This procedure calculates an overall chi-square statistic to test that all of the *D*_*uv*_'s between two markers are zero as follows,

Which has (*k*-1) and (*l*-1) degrees of freedom for markers with *k *and *l *alleles, respectively. A Monte Carlo estimate of the exact *P*-value for testing the hypothesis was calculated by conditioning on the haplotype counts. The significance level is obtained by permuting the alleles at one locus to form 2 *n *new two-locus haplotypes. We used 10,000 permutations to estimate the exact *P-*values.

We used the squared correlation coefficient (*r*^2^) as the LD measure for each pair of alleles *M*_*u *_and *N*_*v *_located at loci *M *and *N*, respectively [75],

Where *D *= *p*_11_*p*_12_- *p*_12_*p*_21 _is the LD coefficient, which can be directly estimated using the observed haplotype frequencies  when using the option HAPLO = GIVEN with the ALLELE procedure of SAS^®^, version 9.3.1 [70]. Since these measures are designed for biallelic markers, the measures are calculated for each allele at locus *M *with each allele at locus *N*, where all other alleles at each locus are combined to represent one allele. Thus for each allele *M*_*u *_in turn,  is used as the frequency of allele *M*_*u*_, and  represents the frequency of "not *M*_*u*_"; similarly for each *N*_*v *_in turn,  represents the frequency of allele *N*_*v*_, and  the frequency of "not *N*_*v*_."

### Linkage disequilibrium decay with distance

The decline of linkage disequilibrium with distance (recombination rate in Morgans) was estimated by fitting the following equation [76],

Where  is the observed *LD *for marker pair *i *in chromosome *j*, the constant *k *= 2 for sex chromosome and *k *= 4 for autosomes, *d*_*ij *_is the recombination rate from two-point linkage analysis for marker pair *i *in chromosome *j, b*_*j *_is the estimate of effective population size for chromosome *j*, and *e*_*ij *_is a random residual. The estimates of *r*^2 ^for pairs of markers were adjusted for experimental sample size , where *n *is the chromosome sample size (*n *= 192). We performed the non-linear modeling with JMP^® ^Genomics 3.1 (SAS Institute Inc., Carey, NC, 2007).

### Linkage disequilibrium between nonsyntenic loci

We estimated LD adjusted for experimental sample size (*r*^2 ^- ) between pairs of non-syntenic loci using the ALLELE procedure of software package SAS^®^, version 9.3.1 [70]. As input data, we used marker genotype data with alleles recoded into a consecutive-numered system (non-phased marker genotype data). In the analysis, we used the option HAPLO = EST which indicates that the maximum likelihood estimates of the haplotype frequencies are used to calculate the LD test statistic as well as the LD measures. For LD estimation among non-syntenic loci, we used the ALLELE procedure options ALLELEMIN = GENOMIN = HAPLOMIN = 0.01. This last statement ensures that only alleles, genotypes, and haplotypes with frequency ≥ 0.01 are used in the LD analysis.

### Effective population size

The analysis was based on the known relationship between *LD *as measured by *r*^2 ^(squared correlation of allele frequencies at a pair of loci) and effective population size *N*_*e*_,

Where *c *is the recombination rate between the microsatellite loci and *n *is the experimental sample size. The constant *α *= 1 in the absence of mutation [76] and *α *= 2 if mutation is taken into account [77-79]. The constant *k *was set to *k *= 2 for sex chromosome and *k *= 4 for autosomes.

Given the formulae described in the linkage disequilibrium section, and knowing *r*^2 ^and *c*, we estimated *N*_*e *_for each chromosome by fitting this nonlinear regression model,

Where  is the observed LD (adjusted for chromosome sample size *n*) for marker pair *i *in chromosome *j*, *c*_*ij *_is the recombination rate from two-point linkage analysis for marker pair *i *in chromosome *j*. The parameter *β*_*j *_is the estimator of effective population size for chromosome *j *where . The parameters *α*_*j *_and *β*_*j *_were estimated iteratively using non-linear modeling with JMP^® ^Genomics 3.1 (SAS Institute Inc., Carey, NC, 2007).

## Authors' contributions

CR participated in design, marker selection, genotyping, and drafted the manuscript, RV participated in design, performed the calculations for LD and effective population size. Both authors read and approved the final manuscript.
